# Health and Well-Being of International University Students, and Comparison with Domestic Students, in Tasmania, Australia

**DOI:** 10.3390/ijerph15061147

**Published:** 2018-06-01

**Authors:** Sarah Skromanis, Nick Cooling, Bryan Rodgers, Terry Purton, Frances Fan, Heather Bridgman, Keith Harris, Jennifer Presser, Jonathan Mond

**Affiliations:** 1 Centre for Rural Health, University of Tasmania, Launceston 7250, Australia; Sarah.Skromanis@utas.edu.au (S.S.); Terry.Purton@utas.edu.au (T.P.); Heather.Bridgman@utas.edu.au (H.B.); 2 School of Medicine, University of Tasmania, Hobart 7000, Australia; Nick.Cooling@utas.edu.au (N.C.); K.Harris@utas.edu.au (K.H.); Jennifer.Presser@utas.edu.au (J.P.); 3 School of Demography, Australian National University, Canberra 0200, Australia; Bryan.Rodgers@anu.edu.au; 4 School of Education, University of Tasmania, Launceston 7250, Australia; Si.Fan@utas.edu.au; 5 School of Medicine, Western Sydney University, Sydney 2560, Australia

**Keywords:** international students, Australia, health and well-being, help-seeking

## Abstract

International students comprise an increasingly larger proportion of higher education students globally. Empirical evidence about the health and well-being of these students is, however, limited. We sought to examine the health and well-being of international students, primarily from Asian countries, attending the University of Tasmania, Australia, using domestic students as a comparison group. Ethics approval was given to invite (via email) all currently enrolled students to participate in the study by completing a pilot-tested, online survey. The survey was completed by 382 international students (response rate = 8.9%) and 1013 domestic students (9.2%). Independent samples *t*-tests, analysis of variance (ANOVA) and chi-square tests were used for bivariate comparisons between international and domestic students, and between subgroups of international students. Regression models were used to examine the associations between student status (international vs. domestic) and health outcomes, controlling for demographic and enrolment variables. International students, particularly male students, were found to be at increased risk of several adverse health outcomes while also being less likely to seek help for mental health and related problems. The findings indicate the need for accessible, targeted, culturally-sensitive health promotion and early intervention programs.

## 1. Introduction

The proportion of international students enrolling in Australian universities has increased considerably in recent years, such that these students now comprise some 30 percent of the nation’s higher education population [1]. This trend has prompted an increased appreciation for, and awareness of, the challenges and varying needs of international students.

The commencement of tertiary studies is often associated with both personal (e.g., gaining greater social, emotional and financial independence) and structural (e.g., adapting to a new educational environment) challenges [2]. These challenges may, however, be particularly common, and particularly difficult to manage, for international students [3,4]. When relocating to their chosen study location, international students’ social support networks are left behind, and establishing comparable relationships in a new country can be difficult. This is particularly problematic for students moving from collectivist cultures, wherein social cohesion and close relationships are promoted, to countries with more individualistic social structures [5,6].

Inability to effectively manage the acculturation process, coupled with the additional pressures associated with being an international student, such as specified course completion times and pressure to perform well and gain tertiary qualifications, can result in adverse impacts on students’ physical, mental and social well-being [7]. Cemalcilar and Falbo (2008) [8] found, in a sample of international university students in the USA, that relocating to a host country has adverse effects on mental health, even among those who are more familiar with the host culture (i.e., those whose home culture does not largely differ to that of the host culture). Some studies have focused on specific ethnic groups, such as Chinese students in an urban university in Sydney who experienced higher psychological morbidity than local students [9].

In addition to experiencing poorer health and well-being more generally, international students may also be at increased risk of engaging in health compromising behaviours, such as alcohol and substance use and problem gambling behaviours, when compared with domestic students [6,10]. While engagement in such behaviours likely reflects the tendency of young peoples to experiment with risky activities more generally, these behaviours may be particularly appealing to international students as they may be particularly prone to seeking the temporary escape from educational and acculturational stressors that these behaviours afford. For some, the appeal of these activities may be enhanced by the relative ease of access in their chosen study location when compared with their home country [5,6].

In one of the few studies to specifically address the health and well-being of international students in Australia, Rosenthal and colleagues (2008) [10] found that one-third of the sample regarded their health as being only fair, and one-fifth believed that their health was having strong adverse impacts on their studies. In relation to engagement in health compromising behaviours, a concerning minority (1–6% depending on the behaviour) had begun using illicit drugs, tobacco smoking or gambling, and one-quarter reported an increase in their alcohol use since relocating to Australia. Comparisons between international and domestic students on these outcomes were not conducted in this study. These findings highlight the potential role of acculturative stressors in increasing international students’ vulnerability to negative health consequences.

Of interest is that in both Rosenthal et al.’s (2008) [10] and Moore et al.’s (2013) [6] studies, the occurrence of at least some health compromising behaviours was found to be more common among male international students than among female international students. Thus, in Rosenthal et al.’s (2008) [10] study, both problem gambling behaviour and tobacco use were found to be more common among male students than female students. Similarly, in Moore et al.’s (2013) [6] study, problem gambling behaviours were found to be significantly more common among male international students (9.7%) than among female international students (3.9%). These findings raise the possibility that the health and well-being of male international students, in particular, may warrant attention in campus-based health promotion and early intervention programs.

Despite the concerning proportion of international students who report high levels of psychological distress and frequent engagement in health-compromising behaviours following relocation to Australia, little research has examined the help-seeking behaviours of these students. Russell, Rosenthal and Thomson (2009) [11], however, examined the help-seeking behaviours of 979 international students at a large Australian university and found that 37.8 percent of these students had felt a need to seek help, but had chosen not to do so. Among these students, the most frequently reported reason for not seeking help was believing that their problem was not important enough, followed by a lack of knowledge about available services. There was no comparison with domestic students in this study, however. Similar findings were also reported by Eisenberg, Downs, Golberstein and Zivin (2009) [12] who found that male international students, across 13 universities in the USA, were particularly unlikely to seek help for mental health-related issues compared with domestic students. Possible reasons for this, in addition to those mentioned above, may include stigmatisation of certain health services, norms surrounding privacy and disclosure, language barriers and beliefs about the cross-cultural competence of health care providers [10,13]. These findings, coupled with those suggesting poorer general health and well-being among international students, highlights the importance of not only providing culturally-sensitive support services, but also demystifying and normalising the help-seeking process.

In sum, while the health and well-being of international students is increasingly becoming of interest in Australia and elsewhere, evidence in this regard remains limited. The aim of this study was to expand the current evidence base by examining the health and well-being of international students at an Australian university, and to compare outcomes between international and domestic students. Aspects of health and well-being assessed included self-rated general and mental health, social support, environmental satisfaction, the occurrence of various health-compromising behaviours and help-seeking for mental health and related problems. Based on the available evidence, it was hypothesised that international students would have poorer health and well-being than domestic students on at least some of the above-mentioned outcomes. Additionally, we hypothesised that international students would be less likely to seek help for mental health and related problems than domestic students. We also considered sex differences in health and well-being outcomes among international students. In this regard, we hypothesised, first, that the health and well-being of male international students would be poorer than that of female international students on at least some outcome measures; and second, that male international students would be less likely to seek help for mental health and related problems than female international students.

## 2. Materials and Methods 

### 2.1. Study Setting

Tasmania, an island state lying to the south of the mainland, is a relatively sparsely populated, primarily rural and semi-rural region of Australia. It has a population of approximately 520,000, two-thirds of whom live outside the state capital city of Hobart (population of approximately 220,000). The study population were international and domestic undergraduate and postgraduate students from a single university in Australia—The University of Tasmania (UTas). UTas has two major campuses in Hobart and Launceston (population of approximately 90,000).

### 2.2. Procedure and Recruitment of Participants

This survey was nested in a larger survey whose purpose was to examine the gambling behaviours and impacts in undergraduate and postgraduate students at one Australian university. Findings from this other component of the research will be reported elsewhere. Data were collected by means of an anonymous, online survey developed for the current research. A pilot study, involving focus groups and completion of hardcopy surveys, was conducted with a convenience sample of international and domestic students to assess the utility of the survey instrument, elicit feedback regarding the survey content and to estimate likely response rates. Minor modifications to the survey instrument were made as a result.

For the main study, all currently enrolled international (*n* = 4289) and domestic (*n* = 10,970) students were invited to participate via a bulk email. This was followed by a single reminder email and a single SMS. The study was also advertised on social media platforms and through the distribution of flyers and postcards at on-and-off campus locations known to be frequented by international students in particular. These methods were employed to maximise response rates from the outset [14]. Following survey completion, which took approximately 20 min, participants were eligible to enter a draw for one of several gift vouchers. To preserve participant anonymity, contact details for the prize draw were obtained through a separate questionnaire from which links to their main questionnaire responses could not be made. Participants were informed that consent was implied by completion of the survey. Students who wished to participate, but were not proficient in English, were invited to contact the researchers for alternative arrangements to be made. There were no students who requested assistance in this regard. Ethics approval to conduct the survey was obtained from the Human Research Ethics Committee at the University of Tasmania.

### 2.3. Measures

#### 2.3.1. Sociodemographic Characteristics

The sociodemographic characteristics assessed included, in addition to student status (international, domestic), age, sex, country of birth, main language spoken at current residence (English, other), marital/relationship status, children, main source of income, enrolment faculty, level of study (undergraduate, postgraduate) and study load (full time, part time).

#### 2.3.2. Mental Health

Mental health was assessed using the 10-item Kessler Psychological Distress Scale (K-10) [15], a brief measure designed to assess levels of psychological distress, based on feelings experienced within the preceding 30 days (e.g., “during the last 30 days, about how often did you feel nervous?”). Total scores ranging from 10 to 50 were calculated, with higher scores indicating higher levels of distress, and scores of 30 or higher being indicative of clinical levels of distress. The K-10 has demonstrated excellent internal consistency (Cronbach’s alpha = 0.89) as determined from a sample of Australian university students [16]. The Cronbach’s alpha score in the current study was 0.95 for international students and 0.93 for domestic students.

#### 2.3.3. Social Support

Social support was assessed using the Multidimensional Scale of Perceived Social Support (MSPSS) [17], a 12-item measure designed to assess the perceived adequacy of social support in each of the three domains, namely, significant others, family members and friends. Item (and domain) scores ranged from one to five with higher scores indicating greater perceived support. The MSPSS has demonstrated excellent internal consistency for the three subscales (Cronbach’s alpha = Friends: 0.93; Family: 0.92; Significant others: 0.93) as determined from a sample of university students [18]. The Cronbach’s alpha score in the current study was 0.94 for international students and 0.92 for domestic students.

#### 2.3.4. Satisfaction with Living Conditions

Satisfaction with living conditions was assessed using the (8-item) Environmental Health subscale of the World Health Organization Quality of Life Short Form (WHOQOL-BREF) [19], a measure designed to capture perceived satisfaction with living conditions among individuals from diverse cultural backgrounds. Item (and scale) scores ranged from one to five with higher scores indicating greater perceived satisfaction with one’s environment. The WHOQOL-BREF environmental subscale has demonstrated acceptable internal consistency (Cronbach’s alpha = 0.80) [20]. The Cronbach’s alpha score in the current study was 0.84 for both international and domestic students.

#### 2.3.5. Subjective Well-Being/Global Life Satisfaction

Subjective health and well-being was assessed using the Satisfaction with Life Scale [21], a five-item measure designed to provide a brief assessment of the individual’s perceived overall satisfaction with their life. Item (and domain) scores ranged from one to five, with higher scores indicating greater satisfaction. The Cronbach’s alpha score in the current study was 0.87 for international students and 0.89 for domestic students. Additional items were included to assess current levels of stress associated with the students’ study and financial situations.

#### 2.3.6. Substance Use

Substance use, including tobacco smoking, alcohol use and illicit drug use, was assessed using items adapted from the Melbourne “Growing Experience” study [10]. Items (three per behaviour) assessed whether participants regarded their use of each substance as being problematic, whether their use had increased since commencing their tertiary studies at UTas, and whether they regarded their use as being out of control. Items were rated on a 5-point Likert-type scale ranging from 1 (“does not apply to me at all/ever”) to 5 (“applies to me completely/all of the time”). Total scores for each behaviour ranged from three to 15, with higher scores indicative of more problematic use.

#### 2.3.7. Gambling Behaviour

The Canadian Problem Gambling Severity Index (PGSI) [22] was employed to assess gambling severity among participants who reported engaging in any form of gambling during the past 12 months. The PGSI is a 9-item measure consisting of statements about participants’ gambling behaviours (e.g., “have you borrowed money or sold anything to get money to gamble”?) that are rated on a 4-point Likert-type scale where 0 = Never and 3 = Almost always. Scores were summed, yielding a total gambling severity score ranging from 0 to 27. Based on total scores, individuals were categorised into one of four categories, namely, non-problem (0), low-risk (1–2), moderate-risk (3–7) and problem gambler (≥8). The Cronbach’s alpha score in the current study among participants who reported any form of gambling in the past 12 months was 0.85 for international students and 0.87 for domestic students.

#### 2.3.8. Self-Rated General Health

Self-rated general health was assessed using a single item that required participants to rate their overall health during the past four weeks on a 5-point scale, from “excellent” to “poor”. Responses to this item have been found to be strongly predictive of more objective measures of health status, including medical morbidity, health service use and mortality [23]. 

#### 2.3.9. Help-Seeking 

Items assessing help-seeking behaviours, which were developed by the authors for the current study based on previous research [24,25], asked whether participants had ever sought advice or help from a health professional in relation to a mental health problem, alcohol or substance use problem, relationship problem, financial problem or gambling problem. Participants who indicated that they had sought advice or help for one or more of these problems were asked to indicate from whom this advice or help had been sought. Finally, participants who indicated that there was a time when they believed that they needed professional help for one or more of the abovementioned problems, but who had never sought such help, were asked the main reason for not seeking such help. 

#### 2.3.10. Statistical Analysis

Statistical analyses were conducted using IBM SPSS Statistics Version 24. Standard methods, namely, independent samples *t*-tests, analysis of variance (ANOVA) and chi-square tests, were used for bivariate comparisons of outcome variables between international and domestic students, and between male and female international students, with post-hoc analysis as appropriate to identify the source of observed differences. Regression models, i.e., multiple linear regression analysis for continuous outcome variables and binary logistic regression analysis for dichotomous outcome variables, were used to examine the associations between student status (international vs. domestic) and each outcome variable in the total study sample while controlling for demographic characteristics and enrolment variables as outlined above. A significance (alpha) level of *p* ≤ 0.05 was employed for all tests and all tests were two-tailed.

## 3. Results

### 3.1. Demographic and Enrolment Characteristics of Participants

Questionnaires with little (<2%) or no missing data were received from 382 international (27.4%) and 1013 domestic (72.6%) students (total *n* = 1395) aged 18 to 72 years (*M* = 26.58, *SD* = 9.72). The response rates were 8.9% and 9.2% for international and domestic students, respectively. Approximately half (52.2%) of the international students and 36.4% of domestic students were male. The demographic and enrolment characteristics of participants, stratified by student status (international vs. domestic), are shown in Table 1. As can be seen, the most common countries of birth for international students were China, Malaysia, India and Singapore. Small numbers of individuals from some 20 other countries comprised the remaining third of international student participants. As can also be seen, international students were more likely than domestic students to be male; single; not have children; receive financial support from their families, be enrolled in maritime, science, engineering and technology, or business and economics degrees (as opposed to arts and law, education or health degrees); be postgraduate (as opposed to undergraduate); and be enrolled full time (as opposed to part time).

### 3.2. Comparison between International and Domestic Students

Comparisons between international and domestic students on each of the health and well-being outcomes assessed are summarised in Table 2. As can be seen, international students reported significantly poorer global life satisfaction and satisfaction with their environmental circumstances, and significantly lower levels of support from significant others and friends than domestic students. Further, international students reported higher levels of smoking and illicit drug use, and were more likely to be classified as problem gamblers than domestic students. Domestic students were more likely than international students to rate their health as being fair or poor, however. While there were no differences between groups with respect to levels of general psychological distress, study stress or financial stress, international students were less likely than domestic students to report seeking help for a mental health problem, a relationship problem or an alcohol or substance use problem.

International students who had sought advice or help for one or more of the problems assessed were more likely to seek help from university counselling services (47.1% vs. 21.9%) and religious leaders (12.6% vs. 4.2%), and less likely to seek help from a general practitioner (25.3% vs. 57.0%), a psychologist in private practice (18.4% vs. 53.5%) or a community mental health service (5.7% vs. 17.9%), than domestic students (all *p* < 0.01), whereas approaches to a psychiatrist in private practice (6.9% vs. 10.3%) and online/telephone counselling services (10.3% vs. 8.2%) did not differ between groups (both *p* > 0.05).

Over one third of international (35.6%) and close to half (49.5%) of domestic students (χ^2^ = 20.0, *p* < 0.01) reported that, at some point, they had felt the need to seek professional help for one or more of the problems mentioned but chose not to do so. Among international students, the most commonly reported reasons for not seeking help were thinking their problem was not important/serious enough (25.8%), feeling that they could manage the problem on their own (24.2%) and thinking the service would not be able to help them (17.5%). 

### 3.3. Comparison between Male and Female International Students

As can be seen in Table 3, male international students showed poorer outcomes than female international students on several of the outcome variables, including lower levels of global life satisfaction, lower perceived support from friends and significant others, higher levels of smoking and higher levels of problem gambling behaviours (all *p* < 0.05).

As can also be seen, male international students were significantly less likely to seek advice and help for a mental health problem relative to female international students. There were no differences between males and females in help-seeking for relationship problems, alcohol/substance use problems, financial problems or gambling problems.

### 3.4. Multivariable Analysis

The results of the regression analysis indicated that, for most of the measures on which differences between international and domestic students were observed with a bivariate analysis—global life satisfaction, support from significant others and friends, illicit drug use, problem gambling behaviours, and help-seeking behaviours—these differences were no longer apparent when controlling for demographic and enrolment characteristics. Satisfaction with environmental circumstances remained poorer (*p* < 0.05), and smoking levels higher (*p* < 0.01), among international students, however, in the multivariable analysis (details available from the first author upon request).

## 4. Discussion

The aim of the current study was to expand the currently limited evidence base by examining the health and well-being of international students in Australia, using domestic students as a comparison group. Aspects of health and well-being assessed included physical and mental health, social support, environmental satisfaction, the occurrence of various health-compromising behaviours, help-seeking behaviours and perceived need for help.

It was hypothesised that international students would have poorer health and well-being, including more frequent engagement in health compromising behaviours, than domestic students. On several outcome measures, there was support for this hypothesis. Thus, international students reported poorer global life satisfaction, poorer perceived social support, greater dissatisfaction with their environmental circumstances, higher levels of smoking and illicit drug use, and higher levels of problem gambling behaviours, than domestic students. Further, and notwithstanding these differences, international students were less likely than domestic students to seek help for mental health and related problems. While more than half of domestic students had sought help for a mental health or related problem, less than one in five international students had sought such help. These findings are consistent with those of a small study at a regional German university [26] demonstrating that international students fared more poorly on health-related outcomes, including psychological distress and perceived social support, relative to their domestic student counterparts. International students were also more likely to engage in health-compromising behaviours, such as smoking, and this was particularly true for male international students.

Of the various outcomes assessed, there was only one outcome for which domestic students fared worse than international students, namely, domestic students were more likely to rate their health as being fair or poor than international students. This finding is somewhat surprising, given the higher levels of smoking and substance use among international students, and likely reflects the influence of variables, such as diet and exercise, not assessed in the current study. In any case, it may be noted that the vast majority of both domestic and international students rated their health as being good, very good or excellent. Self-rated health measures in previous young international student and migrant population studies may have been biased towards the importance of physical health, which tends to be generally more robust in these populations compared with mental health [27,28].

We were also interested to consider sex differences in health and well-being among international students, our hypothesis being that the health and well-being of male international students would be poorer than that of female international students on at least some outcome measures. While there were no outcomes for which females had poorer health, consistent with this hypothesis, male international students reported poorer global life satisfaction, poorer perceived social support and greater engagement in problem gambling behaviours and tobacco use than female international students. Further, and notwithstanding these differences, male international students were less likely than female international students to report ever having sought help for a mental health problem. These findings are consistent with those of a small number of previous studies, including studies of international students in other parts of Australia [6,10], suggesting that male international students may be a particularly vulnerable group in terms of unmet needs for health care.

### 4.1. Study Implications

Since most of the differences between international and domestic students that were apparent in the bivariate analysis, for both health outcomes and help-seeking behaviours, were no longer apparent when between-group differences in demographic and other enrolment variables were statistically controlled for; these differences likely reflect demographic characteristics associated with being an international student, rather than student status per se. In particular, international students participating in the current study were more likely to be male than domestic students (52.2% vs. 36.3%). It is well known that young men, including those engaged in tertiary studies, are more likely to engage in certain health-compromising behaviours, including alcohol and substance use and problem gambling, and less likely to seek help for mental health and related problems, than young women [29], and such differences were observed between male and female international students in the current study. This does not detract from the implication of the current study findings suggested above, however, namely, that male international students may warrant particular attention in campus-based health promotion and early intervention programs. At the same time, it needs to be remembered that programs of this kind are more likely to be effective if they target both male students and those with whom they interact, including their female peers [30,31].

Barriers to help-seeking among international students with mental health and related problems, which need to be addressed in health promotion, early intervention and service delivery initiatives, include poor awareness or understanding of local support services and how to access them, language barriers, and perceived stigma and/or adverse impact on academic progress associated with disclosure of such problems [12,32]. Among international students in the current study, relatively low rates of help-seeking also appeared to reflect, at least in part, lower perceived need for help, since international students (35.6%) were also less likely than domestic students (49.5%) to report feeling a need to seek professional help for a mental health or related problem. Further, the most commonly reported reasons for not seeking help among international students in the current study were thinking that the problem was not important or serious enough and feeling that they could manage the problem on their own. It may be that international students who have already experienced the challenges of relocation, resettling, improving English language skills, and developing social support networks in a new country, regard their health problems as being relatively minor in comparison to these other challenges. Taken together, these findings suggest that improved awareness and understanding of the nature and adverse effects of mental health and related problems may be an important component of interventions designed to improve the uptake of early, appropriate treatment among international students for whom this is needed.

Programs to improve the accessibility of support services to international students, through education and logistic enhancements have been somewhat effective [33]. However, to further improve accessibility, there has been a call for such support services to be more culturally specific and reinforced frequently through the academic year [9,33] In addition, contemporary delivery methods such as internet-based student well-being services appear to provide another solution to the problem of accessibility to support. A recent prototype in an Australian university, *thedesk*, appears to be well accepted by most tertiary students including international students—as long as they are instructed in its use [34]. Also, the building of enduring and meaningful social connections between international students and the host culture has been shown to improve mental health and assist with adjustment processes [35].

### 4.2. Study Limitations and Strengths

At least two limitations of the current research should be noted. First, response rates were low and it is possible that participants differed systematically from non-respondents in certain respects, including aspects of health and well-being. Further, the extent of any such bias may have differed between international and domestic students. While similarly low or lower response rates were observed in the studies of Moore et al. (2013) [6] and Rosenthal et al. (2008) [10], low response rates detract from confidence in the generalisability of the findings. However, with respect to age, sex and country of birth, at least, participants in the current study were representative of international students in Australia as a whole [1]. While the current study was not designed to consider the potential influence of country of birth, or other demographic or enrolment variables in accounting for variance in health-related outcomes among international students, analysis of this kind would be of interest in future research. While a substantially larger sample size would be required to look at the relative importance of all of the demographic or enrolment variables potentially affecting the health and well-being of international students, it would be relatively straightforward to conduct a qualitative study of individual experiences, and this would be a useful adjunct to the quantitative findings reported in the current study.

Second, there was no assessment of international students prior to their relocation. Hence, it is not possible to know whether and to what extent any observed health patterns reflect the influence of events occurring prior to relocation. Information of this kind would clearly have implications for the nature and timing of health promotion and early intervention programs, i.e., whether these programs should focus on existing health differences, monitor deteriorating health status after arrival, or both. Findings from previous research suggest that the occurrence of certain health-compromising behaviours among international students may be precipitated, and exacerbated, by relocation [10].

Additionally, it should be noted that, while a broad range of outcome variables were included in the current study, some important outcomes were not included. For example, there was no assessment of body dissatisfaction or eating-disordered behaviours. Given the over-representation of these problems among young women [30,31], it is likely that female international students would have fared more poorly than male international students on these outcomes, had they been included.

The strengths of the current study were the recruitment of a relatively large sample of male and female international students, the assessment of a broad range of aspects of health and well-being using established measures of these constructs and the inclusion of a comparison group of domestic students. Additionally, while participants in the current study were recruited from metropolitan areas, the populations of these areas were relatively small, and Tasmania is a primarily rural and regional area serviced by a single university. Hence, the current findings extend those of Moore et al. (2012) [6] and Rosenthal et al. (2008) [10], both of which were conducted in a major urban city serviced by several universities. The fact that the current study findings were largely consistent with those of these previous studies, despite the different study settings, supports the generalisability of the findings.

## 5. Conclusions

The current findings contribute to the small existing body of literature on the health and well-being of international university students. They suggest that these students—particularly male students—are at increased risk of several adverse health outcomes while also being less likely to seek help for mental health and related problems when needed. The findings indicate the need for, and will inform, accessible, targeted, culturally sensitive health promotion and early intervention programs in this vulnerable population.

## Figures and Tables

**Table 1:** Demographic and enrolment characteristics of study participants by student status.

Demographic/Enrolment Variables	International (*n* = 382)	Domestic (*n* = 1013)	*t*	*p*
	*M* (*SD*)	*M* (*SD*)		
**Age (years)**	25.3 (5.0)	27.0 (10.9)	4.0	<0.01
	**%**	**%**	**χ^2^**	***p***
**Gender**				
Male	52.4	36.6	28.3	<0.01
Female	47.6	63.4		
**Country of birth**				
Australia	-	93.4	1035.5	<0.01
Great Britain	-	3.6		
China	32.7	1.5		
Malaysia	21.2	0.5		
India	6.8	0.8		
Singapore	5.5	0.2		
**Main Language**				
English	78.1	96.9	129.9	<0.01
Other	21.9	3.1		
**Relationship status**				
Single, never married	75.7	61.9	31.4	<0.01
Single, previously married	1.0	4.5		
Married/living as married	20.7	26.1		
Other	2.6	7.5		
**Children**				
Yes	8.0	18.5	22.7	<0.01
No	92.0	81.5		
**Source of income**				
Paid employment	6.4	40.7	518.8	<0.01
Government pension	1.1	32.0		
University scholarship	13.0	7.1		
Superannuation	1.3	0.8		
Assistance from family	74.5	16.1		
Other	3.7	3.4		
**College/Faculty**				
Australian Maritime College	13.6	2.0	270.2	<0.01
Arts and Law	8.1	21.5		
Education	1.8	11.9		
Health	12.8	31.1		
Science, Engineering & Technology	27.2	17.3		
Business and Economics	24.6	6.9		
Other	11.8	9.2		
**Level of study**				
Undergraduate	50.0	83.9	165.2	<0.01
Postgraduate	50.0	16.1		
**Study load**				
Full time	98.7	82.5	63.7	<0.01
Part time	1.3	17.5		

**Table 2:** Health and well-being by student status.

Health and Well-Being Variables	International Students (*n* = 382)	Domestic Students (*n* = 1013)	*t*	*p*
	*M* (*SD*)	*M* (*SD*)		
Psychological distress ^i^	20.6 (8.7)	20.9 (8.3)	0.7	0.51
Global life satisfaction ^ii^	3.2 (0.8)	3.5 (0.9)	5.4	<0.01
Satisfaction with environment ^iii^	3.6 (0.7)	4.1 (0.6)	12.2	<0.01
**Social support ^iv^**				
Significant others	3.6 (1.2)	4.0 (1.2)	6.0	<0.01
Family	3.9 (0.9)	4.0 (1.0)	1.9	0.05
Friends	3.7 (1.0)	3.9 (1.1)	3.0	<0.01
**Substance use ^v^**				
Tobacco	4.1 (2.5)	3.5 (1.9)	−3.6	<0.01
Alcohol use	4.0 (2.1)	4.3 (1.9)	1.8	0.07
Illicit drug use	3.5 (1.7)	3.3 (1.0)	−2.1	<0.05
	**%**	**%**	**χ^2^**	***p***
**Health and well-being ^vi^**				
Poor health	5.7	10.9	8.0	<0.01
**Study stress ^vii^**				
Stressed	23.2	24.6	0.3	0.61
**Financial stress ^viii^**				
Stressed	19.5	16.9	1.1	0.29
**Problem gambling**				
Non-problem	49.2	66.1	16.3	<0.01
Low-risk	29.2	21.3		
Moderate-risk	13.8	9.8		
Problem	7.7	2.7		
**Help seeking for…**				
Mental health problem	17.1	51.4	124.9	<0.01
Relationship problem	5.3	14.0	18.9	<0.01
Alcohol/substance use problem	0.0	3.0	10.7	<0.01
Financial problem	6.7	4.1	3.9	<0.05
Gambling problem	0.8	0.5	0.4	0.52
**Any**	24.4	55.3	99.2	<0.01

Note: ^i^ Levels of general psychological distress as measured by the *Kessler Psychological Distress Scale* (higher scores indicate greater distress) [15]; ^ii^ Global life satisfaction (subjective well-being) as measured by the *Satisfaction with Life Scale* (higher scores indicating greater satisfaction) [21]; ^iii^ Satisfaction with environmental conditions as measured by the *World Health Organization Quality of Life Short Form (WHOQOL-BREF Environment subscale)* (higher scores indicate greater satisfaction) [19]; ^iv^ Perceived support from significant others, family member and friends as measured by the *Multidimensional Scale of Perceived Social Support* (higher scores indicate greater perceived support) [17]; ^v^ Levels of smoking, alcohol and substance use as measured by items of the Melbourne “Growing Experience” study (higher scores indicate higher levels of the behaviours concerned) [10]; ^vi^ Poor health refers to the proportion of participants who selected either “fair” or “poor”; ^vii^ Study stress refers to the proportion of participants who selected either “very stressed” or “extremely stressed”; ^viii^ Financial stress refers to the proportion of participants who selected either “very stressed” or “extremely stressed”.

**Table 3:** Health and well-being among international students by sex.

Health and Well-Being Variables	Males (*n* = 199) ^1^	Females (*n* = 181) ^1^	*t*	*p*
	*M* (*SD*)	*M* (*SD*)		
Psychological distress	19.8 (9.1)	21.3 (8.2)	−1.7	0.10
Global life satisfaction	3.2 (0.8)	3.3 (0.8)	−2.1	<0.05
Satisfaction with environment	3.6 (0.7)	3.5 (0.6)	1.2	0.25
**Social support**				
Significant others	3.5 (1.2)	3.7 (1.1)	−2.4	<0.05
Family	3.8 (1.0)	4.0 (0.9)	−1.3	0.21
Friends	3.5 (1.0)	3.8 (0.9)	−2.8	<0.01
**Substance use**				
Tobacco use	4.4 (2.9)	3.7 (1.9)	2.8	<0.01
Alcohol use	4.0 (2.1)	4.0 (2.0)	−0.0	1.0
Illicit drug use	3.5 (1.8)	3.4 (1.6)	0.8	0.46
	**%**	**%**	**χ^2^**	***p***
**Health and well-being**				
Poor health	3.4	7.6	2.9	0.09
**Study stress**				
Stressed	20.8	26.0	1.3	0.25
**Financial stress**				
Stressed	17.6	21.6	0.9	0.35
**Problem gambling**				
Non-problem	40.3	58.1	12.0	<0.01
Low-risk	28.4	30.6		
Moderate-risk	16.4	11.3		
Problem	14.9	0.0		
**Help seeking for…**				
Mental health problem	11.7	22.9	7.7	<0.01
Relationship problem	3.4	6.9	2.3	0.13
Alcohol/substance use problem	0.0	0.0	-	-
Financial problem	5.0	8.0	1.3	0.26
Gambling problem	1.1	0.6	0.3	0.58
**Any**	17.3	31.4	9.6	<0.01

Note: ^1^ These figures do not sum to N, as two participants chose not to indicate their sex or indicated that they were “intersex” or “other”.
